# Anonymity for Young Criminals

**Published:** 1921-03-26

**Authors:** 


### Anonymity for Young Criminals.

It is generally admitted (writes a correspondent, apropos a recent article in these columns on the prevention of crime) that dealing with young law-breakers in special juvenile courts instead of in the ordinary police-court is an improvement on our former methods of administering justice; but in Canada authority has taken a still further step. There no reports are issued of the trials of child criminals, and reporters are excluded from the court, although the clerk to the court may, if he chooses, tell of the cases if the names are suppressed. There is much to be said for this reticence. Child criminals are often only high-spirited youngsters, who in another social sphere would be dealt with by schoolmasters instead of the police, and it is hard that their delinquencies should be made public, to be brought up against them in the future. Harder, perhaps, for their parents than for themselves, for while the children themselves may possibly be inclined to " swank " about their notoriety (and that is a bad thing), the parents are sure to be humiliated. Nevertheless, the exclusion of reporters is not entirely a good thing. It is not every man who is the heaven-born judge of a juvenile court. We read of unreasonably harsh sentences inflicted on children; and public opinion, to which the newspaper acts as a conductor, may be of great value in controlling the hardness of a man who has forgotten his childhood and its pranks. There are, unfortunately, judges who have as little compunction in sending a boy to a reformatory for five years because he robbed an orchard as they would have in sending a man to penal servitude for the same period because he had systematically swindled hundreds of his fellow-men. Therefore some outside influence is needed. In Montreal, we learn from Mr. John Kidman, Hon. Secretary of the Canadian Prisoners' Welfare Association, the difficulty is met by the institution of a Juvenile Court Committee, which is consulted on every case before judgment is pronounced. Such a committee, composed of men and women, is likely to have a moderating influence on the verdict. They could always inquire into the young criminal's environment and decide whether or not he should be removed from its influence. Failing such a committee, a report written as picturesquely as may be, but without giving names or any hint of the individuality of the offender, might be very useful in drawing public attention to those whom a very little may either bring back to the paths of honesty or turn into confirmed criminals.

